# How can the occurrence of delayed elevation of thyroid stimulating hormone in preterm infants born between 35 and 36 weeks gestation be predicted?

**DOI:** 10.1371/journal.pone.0220240

**Published:** 2019-08-23

**Authors:** You Jung Heo, Young Ah Lee, Bora Lee, Yun Jeong Lee, Youn Hee Lim, Hye Rim Chung, Seung Han Shin, Choong Ho Shin, Sei Won Yang

**Affiliations:** 1 Department of Pediatrics, Seoul National University Children’s Hospital, Seoul National University College of Medicine, Seoul, Korea; 2 Institute of Environmental Medicine, Seoul National University Medical Research Center, Seoul, Korea; 3 Department of Pediatrics, Seoul National University Bundang Hospital, Seongnam, Korea; Holbæk Hospital, DENMARK

## Abstract

**Objective:**

We evaluated frequency and risk factors of delayed TSH elevation (dTSH) and investigated follow-up outcomes in the dTSH group with venous TSH (v-TSH) levels of 6–20 mU/L according to whether late preterm infants born at gestational age (GA) 35–36 weeks had risk factors.

**Methods:**

The medical records of 810 neonates (414 boys) born at Seoul National University Hospital who had a normal neonatal screening test (NST) and underwent the first repeat venous blood test at 10–21 days post birth were reviewed.

**Results:**

Seventy-three (9.0%) neonates showed dTSH, defined as a v-TSH level ≥6.0 mU/L, 12 of whom (1.5%) were started on levothyroxine medication. A multivariate-adjusted model indicated that a low birth weight (LBW <2,000 g), a congenital anomaly, and exposure to iodine contrast media (ICM) were significant predictors for dTSH (all p < 0.05). Among these 73 dTSH infants, all 5 infants with TSH levels ≥20 mU/L began levothyroxine medication, and 6 of 16 infants with v-TSH levels of 10–20 mU/L were indicated for levothyroxine, regardless of coexisting risk factors. However, only 1 of 52 infants with v-TSH levels of 6–10 mU/L who had a congenital anomaly was indicated for levothyroxine. All healthy late preterm infants, including LBW and multiple births, with v-TSH levels of 6–10 mU/L exhibited normal thyroid function.

**Conclusions:**

dTSH was detected in 9.0% and levothyroxine was indicated in 1.5% of infants born at GA 35–36 weeks, particularly those with a LBW, a congenital anomaly, or history of ICM exposure. Either levothyroxine or retesting is indicated for late preterm neonates with TSH levels ≥10 mU/L regardless of risk factors. If healthy preterm neonates show v-TSH levels of 6–10 mU/L, a second repeat test may not be necessary; however, further studies are required to set a threshold for retesting.

## Introduction

Congenital hypothyroidism (CH) is a cause of neurocognitive impairment that is preventable when detected early using neonatal screening tests (NSTs) and treatment [1]. However, some neonates exhibit significantly elevated levels of thyroid-stimulating hormone (TSH) at later than 2 weeks after birth, despite having had normal NST results. As such, repeated thyroid screening is recommended for neonates deemed to be at high risk of delayed thyroid dysfunction. According to the European society for paediatric Endocrinology consensus guidelines published in 2014 (ESPE guidelines), a second screening is recommended for preterm neonates <37 weeks of gestational age (GA), those with low or very low birth weight (LBW or VLBW), critically ill infants admitted to a neonatal intensive care unit (NICU), and multiple-birth neonates (particularly same-sex twins) at around 2 weeks after birth, or 2 weeks after the first screening test [2]. The Japanese Society for Pediatric Endocrinology (JSPE) also recommends a second screening for premature neonates and birth weight (Bwt) <2,000g infants, even if the results of the first screening are within the normal range [3].

The possible mechanisms of delayed thyroid dysfunction are diverse and complex, and include immaturity of the hypothalamus pituitary thyroid (HPT) axis, especially in acutely ill preterm infants; infusions of dopamine, high-dose glucocorticoids, and/or antibiotics [4–7]; exposure to iodine contrast media (ICM) as a result of diagnostic imaging or therapeutic intervention or to disinfectant containing iodine [8, 9]; a congenital anomaly and/or genetic syndrome [10, 11]; exposure to maternal antithyroid drugs and/or TSH receptor-blocking antibody [12]; and LBW itself [13, 14].

The levels of TSH and free thyroxine (fT4) detected in a second screening test may determine the necessity of follow-up testing and treatment, as well as clinical status. Where venous TSH (v-TSH) level is ≥20 mU/L, treatment is indicated regardless of venous fT4 (v-fT4) level; however, in healthy neonates with normal v-fT4 levels who exhibit v-TSH levels of 6–20 mU/L in beyond 3 weeks, the treatment strategy is still debated among clinicians. The natural course of infants with v-TSH levels of 6–20 mU/L needs to be evaluated to determine whether to initiate treatment immediately, retest after 2 weeks, or end repeated testing.

Although the reference TSH range has been proposed as 1.7–9.1 mU/L for neonates between 2 and 6 weeks of age [12, 15], there has been no consensus regarding the recommended TSH levels at a second screening test to complete repeated screening and ensure that patients with CH who may require medication are not overlooked. In particular, as most healthy late preterm neonates have normal thyroid function, the cutoff TSH levels need to be investigated to complete repeated screening tests.

We evaluated the frequency and risk factors for delayed TSH elevation (dTSH) and investigated follow-up outcomes in the dTSH group with v-TSH levels of 6–20 mU/L according to whether late preterm infants born at GA 35–36 weeks had risk factors.

## Subjects and methods

This study was approved by the institutional ethics committee of the Seoul National University Hospital, and a waiver of documentation of consent was granted (IRB No. 1801-132-917). The medical records of preterm infants born in Seoul National University Hospital (SNUH) were retrospectively reviewed. In total, between August 2014 and January 2018, 1,145 infants were born at GA 35–36 weeks and admitted to the newborn ward (NB, n = 917) and the NICU (n = 228). We initially included 1,085 neonates who had undergone NST within 7 days after birth in SNUH. After excluding 260 infants whose parents sought second screening tests outside the hospital, 825 infants underwent the first repeat venous blood test as part of a second screening at SNUH. Fifteen infants with abnormal NST results, including TSH ≥12 mU/L, were also excluded. Finally, 810 infants (648 from NB and 162 from NICU) were included in this study (S1 Fig).

Demographic data were collected regarding sex, birth history, including GA; Bwt; natural or *in vitro* fertilization (IVF) pregnancy, singleton or multiple birth (twin or triplet birth), chorionicity of placenta detected on fetal sonography prior to birth, NICU admission, vaginal or caesarian delivery, maternal age during pregnancy, the presence of maternal thyroid disease, the presence of congenital heart disease (excluding patent foramen ovale or patent ductus arteriosus) and/or other congenital anomalies; diagnosis with chromosomal disorders or genetic syndrome, and exposure to ICM and/or disinfectant containing iodine after surgery.

**Fig 1:**
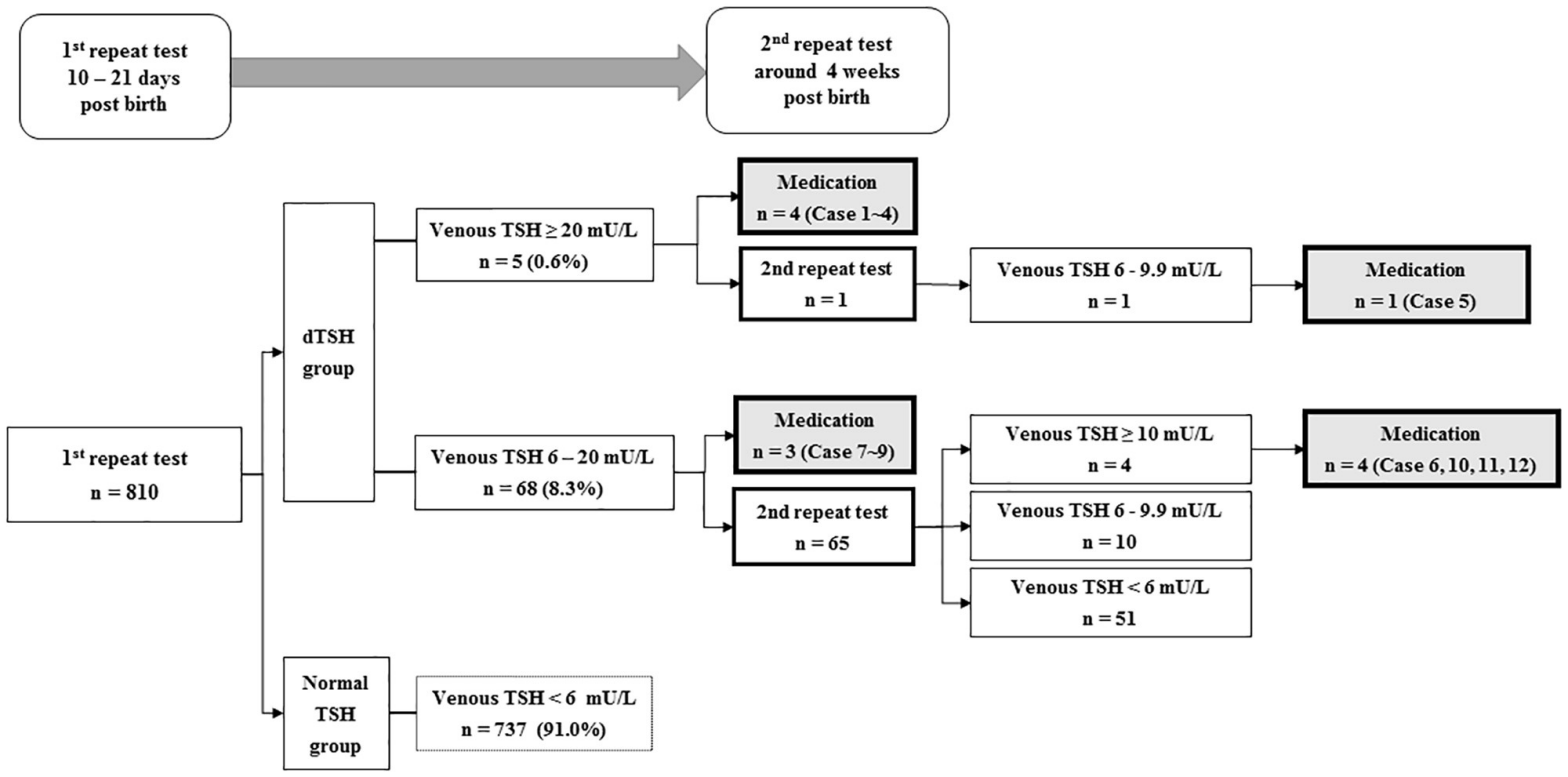
Follow-up results of a second thyroid screening test in preterm neonates born at gestational ages (GAs) 35 and 36 weeks. The first repeat test was at around 2 weeks (10–21 days) post birth and the second repeat test was at around 4 weeks post birth.

All neonates underwent NST via the heel-prick test using a chemiluminescence immunoassay (Modular Analysis E 170 module, Roche, Mannheim, Germany), and the results revealed normal TSH levels <12 mU/L. All repeat screening tests were completed with venous blood samples. Independent of normal NST results, for all late preterm neonates, the first repeat venous blood test was conducted between 10 and 21 days post birth according to the 2014 ESPE guidelines [2]; if the results of serum v-TSH levels were ≥6.0 mU/L, a second repeat venous blood test was performed at around 4 weeks post birth. Venous concentrations of fT4 and TSH were measured using immunoradiometric kits (RIAKEY; Shin Jin Medics, Seoul, Korea). The normal range of v-fT4 is 0.70–1.80 ng/dl (9.01–23.2 pmol/l). Infants with v-TSH values ≥6 mU/L at the first repeat test were categorized into the dTSH group (n = 73, Fig 1). Based on the follow-up results, the dTSH group was further subcategorized into the eventually normalized TSH group (n = 61) and the levothyroxine-treated group (n = 12, Table 1) according to whether the infants had begun receiving levothyroxine medication.

**Table 1:** Comparison of clinical and biochemical characteristics between the normal TSH and delayed TSH elevation groups.

	Total (n = 810)	Normal TSH group (n = 737)	Delayed TSH elevation group		
			Total (n = 73)	Eventually normalized group (n = 61)	Levothyroxine-treated group (n = 12)
Boys, n (%)	414 (51.5)	375 (50.9)	39 (53.4)	30 (49.2)	9 (75.0)
Birth weight (g)	2302.9 ± 390.6	2314.8 ± 376.5	2180.3 ± 499.2^a^	2171.3 ± 468.7	2225.8 ± 656.2
Low birth weight (<2,000 g), n (%)	170 (21.0)	146 (19.8)	24 (32.9)^b^	20 (32.8)	4 (33.3)
NICU admission, n (%)	162 (20.0)	133 (18.0)	29 (39.7)^c^	20 (32.8)	9 (75.0)
Multiple birth, n (%)	672 (83.0)	620 (84.1)	52 (71.2)^b^	45 (73.8)	7 (58.3)
Singleton/twin/triplet birth, n (%)	138/491/181 (17.0/60.6/22.3)	117/446/174 (15.9/60.5/23.6)	21/45/7 (28.8/61.6/7.6)^d^	16/39/6 (26.2/63.9/6)	5/6/1 (41.7/50.0/8.3)
Monochorionicity, n (%)	130 (16.0)	118 (16.0)	12 (16.4)	9 (14.8)	2 (16.7)
Caesarian delivery, n (%)	531 (65.6)	492 (66.8)	39 (53.2)^a^	31 (50.8)	8 (66.7)
IVF pregnancy, n (%)	532 (65.7)	488 (66.2)	44 (60.3)	39 (63.9)	5 (41.7)
Maternal age at pregnancy (years)	34.0 ± 3.5	34.0 ± 3.5	34.4 ± 4.2	34.5 ± 4.4	33.7 ± 3.0
Maternal thyroid disease, n (%)	111 (13.7)	98 (13.3)	13 (17.8)	12 (19.7)	1 (8.3)
Congenital heart disease excluding PFO or PDA, n (%)	55 (6.8)	42 (5.7)	13 (17.8)^b^	8 (13.1)	5 (41.7)
Other congenital anomalies, n (%)	48 (5.9)	31 (4.2)	17 (23.3)^c^^,^ ^f^	12 (19.7)	5(41.7)
Exposure to iodine contrast media, n (%)	12 (1.5)	2 (0.3)	10 (13.7)^c^	6 (9.8)	4 (33.3)
History of surgery, n (%)	12 (1.5)	7 (0.9)	5 (6.8)^b^	3 (4.9)	2 (16.7)
TSH levels at NST (mU/L)	4.7 ± 2.1	4.5 ± 2.0	6.6 ± 2.7^c^	6.1 ± 0.2	8.9 ± 2.5
First repeat venous fT4 (ng/dl)	1.2 ± 0.2	1.2 ± 0.2	1.2 ± 0.2	1.3 ± 0.2	1.1 ± 0.3
First repeat venous TSH (mU/L) ^e^	3.5 ± 1.6	2.7 ± 1.4	10.9 ± 12.6^c^	8.0 ± 1.9	25.8 ± 27.0

Data are mean ± standard deviation (SD) or number (%).

TSH, thyroid stimulating hormone; NICU, neonatal intensive care unit; IVF, *in vitro* fertilization; PFO, patent foramen ovale; PDA, patent ductus arteriosus; NST, newborn screening test; fT4, free T4

^a^ *P* < .05 using the χ2 test or Student’s *t*-test vs. the normal group

^b^ *P* < .01 using the χ2 test or Fisher’s exact test vs. the normal group

^c^ *P* < .001 using the χ2 test, Fisher’s exact test, or Student’s *t*-test vs. the normal TSH group

^d^ *P* < .001 using the χ2 for trend among three groups

^e^ Log-transformed

^f^ Epidermolytic hyperkeratosis, vertebral anomaly, jejunal atresia, bilateral cleft palate and lip, neonatal cholestasis (2), coloboma, congenital dural arteriovenous fistula combined with hydrocephalus, imperforated anus, omphalocele, hereditary hearing loss, inguinal hernia, congenital pulmonary airway malformation and sequestration, biliary atresia, sacral dimple, filar cyst, undescended testis.

Levothyroxine replacement was strongly indicated when v-TSH levels were ≥20 mU/L and/or v-fT4 levels were <0.7 ng/dl at the first repeat test. Where levothyroxine treatment had been indicated for an infant, the starting dose per body weight and duration of treatment were reviewed. When v-TSH levels were between 6 and 20 mU/L, and v-fT4 levels were within the normal range, we decided, after a discussion with the parents, to either commence treatment with levothyroxine or retest 2 weeks later without administering any treatment. If v-TSH levels remained higher than 10 mU/L at up to 4 weeks post birth, we suggested starting levothyroxine treatment. Infants whose v-TSH levels persistently measured between 6 and 10 mU/L were followed-up without receiving medication, until TSH levels normalized (Fig 1).

Statistical analyses were performed using IBM SPSS Statistics ver. 22.0 (IBM Co., Armonk, NY, USA) and R version 3.5.2 (The Comprehensive R Archive Network, Vienna, Austria; http://cran.r-project.org). All continuous variables were described as means ± SD. Variables were tested for a normal distribution. Variables with a skewed distribution were log-transformed for analysis. Student’s *t*-test was used to compare the means of continuous variables with normal distribution between the two groups. The chi-squared test and Fisher’s exact test were used to compare categorical variables between the two groups. The chi-square test for trend analysis was performed to compare categorical variables among the three groups. Odds ratios were obtained by performing univariate and multivariate logistic regression analyses to identify independent risk factors for occurrence of dTSH. In multivariate models, we investigated model stability by assessing the importance of variables and the proportion of variance explained by each model using 100 bootstrap replications. The bootstrapping was conducted using generalized linear models to predict the occurrence of dTSH based on eight predictors (LBW, NICU admission, multiple birth, caesarian delivery, congenital heart disease, other congenital anomalies, exposure to ICM, and history of surgery). Specifically, to test the selection of variables in the models, we compared a −2 × log-likelihood estimate (a measure of description loss against model complexity) with or without each covariate from among eight predictors in a model of 100 resampled data using *mplot* in R [16]. In addition, we assessed model stability by comparing R^2^ for the proportion of variance explained by the models with eight predictors in each permutation [17]. Statistical significance was defined as *P* < 0.05.

## Results

### Baseline characteristics and frequency of dTSH

The clinical characteristics of the 810 infants (414 boys and 396 girls) are summarized in Table 1. Mean GA was 35.9 weeks (0.7 of SD), and mean Bwt was 2,302 g (390.6 of SD, range 1,060–5,050 g). Bwt <2,000 g infants numbered 170 (21.0%). A total of 162 (20.0%) infants were admitted to the NICU. As our hospital is a tertiary referral center where mainly high-risk pregnant women are transferred, multiple births accounted for 672 (83%) of births; 130 (16.0%) of these were monochorionic twins. Caesarian sections accounted for 531 (65.6%) of all births, and 532 (65.7%) infants were born following an IVF pregnancy. Mean maternal age at pregnancy was 34.0 years (3.5 of SD), and 111 (13.7%) mothers had a history of thyroid disease. Infants with congenital heart disease numbered 55 (6.8%), while congenital anomalies were detected in 48 (5.9%) infants. Twelve (1.5%) had been exposed to ICM, and 12 (1.5%) had undergone surgery. Among 672 infants born as multiple births, twins and triplets accounted for 491 and 181 births, respectively. As the number of offspring in a birth increased from singleton to twin to triple births, the proportions of LBW (p < 0.001), caesarian delivery (p < 0.001), and IVF pregnancy (p < 0.001) among the study subjects increased; however, the proportions with congenital heart disease (p < 0.001), congenital anomalies (p < 0.001), exposure to ICM (p = 0.002), and history of surgery (p = 0.002) decreased (S1 Table).

Mean age at NST was 3.7 days (1.3 of SD). The first and second repeat blood tests were performed at 15.2 days (2.5 of SD) and 31.1 days (9.8 of SD) post birth, respectively. dTSH was detected in 73 (9.0%) of 810 infants (Table 1 and Fig 1); these were further subcategorized into the eventually normalized TSH group (n = 61) and the levothyroxine-treated group (n = 12, 1.5%; Table 2, and gray solid box in Fig 1). Among 73 infants with dTSH, 52 were multiple births (45 twins and 7 triplets), seven of whom began receiving levothyroxine (Table 1). None exhibited transient hypothyroxinemia of prematurity, defined as low v-fT4 and normal v-TSH levels. However, two infants (cases 1 and 6 in Table 2) showed low v-fT4 (<0.7 ng/dL) and significantly elevated v-TSH levels, implying primary hypothyroidism and requiring prompt levothyroxine supplementation.

**Table 2:** Characteristics of the 12 patients who received levothyroxine treatment.

No	GA (wks)	Birth weight (g)	Sex	Delivery mode	ART	Plurality	Clinical characteristics	First screening test (NST)		Second screening test (venous blood sample)						Treatment (levothyroxine) Dose and duration
										First repeat test			Second repeat test			
								day	TSH	day	fT4	TSH	day	fT4	TSH	
1	36 + 0	2810	M	C/S	IVF	Singleton	Omphalocele, congenital nephrotic syndrome	6	6.7	18	0.43	108.4				12.7 μg/kg/day, deceased at 3 months of age
2	35 + 4	1060 (SGA)	M	C/S	IVF	DC twin	Maternal hypothyroidism, neonatal cholestasis, inguinal hernia	6	10.7	13	0.89	31.7				12.1 μg/kg/day, currently taking after 2 years
3	36 + 5	2110	F	C/S	(-)	DC twin	DORV, combined PS, VSD, omphalocele, scoliosis, facial anomaly	7	11.5	16	0.9	26.6				8.6 μg/kg/day, deceased at 3 years of age
4	35 + 6	2400	F	C/S	(-)	DC twin,	PHT	6	11.9	14	1.18	24.6				6.4 μg/kg/day, discontinued after 2 weeks
5	35 + 2	1310	M	C/S	IVF	MC triplet	Exposure to ICM due to meconium obstruction	7	10	14	1.5	21.7	23	1.09	8.1	3.1 μg/kg/day, starting at 150 days old and currently taking after 14 months
6	36 + 1	2580	F	Vag	(-)	Singleton		2	8.7	15	0.77	18.5	22	0.65	33.4	13.3 μg/kg/day, currently taking after 18 months
7	35 + 5	3170	M	C/S	IVF	Singleton	Jejunal atresia, exposure to ICM for loopogram	6	11.7	20	1.33	17.9				7.0 μg/kg/day, currently taking after 2 years.
8	36 + 3	2460	M	Vag	(-)	Singleton	TOF, PA, exposure to ICM for cardiac CT (26days of birth)	6	3.6	13	1.2	16.3				5.3ug/kg/day, discontinued after 15 months
9^a^	36 + 0	1950 (SGA)	M	Vag	(-)	MC twin		2	7.3	21	1.02	14.1				9.6 μg/kg/day, discontinued after 12 months
10	36 + 3	1490 (SGA)	M	Vag	IVF	DC twin	VSD, PHT, vertebral anomaly	6	9.4	16	1.35	12.8	22	1.17	13.5	7.9 μg/kg/day, discontinued after 8 months
11 ^a^	36 + 0	2540	M	C/S	(-)	MC twin		6	9.4	14	1.17	10.5	23	1.13	16.2	7.9 μg/kg/day, discontinued after 2 months
12	36 + 0	2830	M	C/S	(-)	Singleton	Valvar PS, PHT, congenital dural AVF, hydrocephalus, exposure to ICM for brain MRI (6 days after birth)	6	8.8	12	1.24	6.3	34	0.85	47.5	10.6 μg/kg/day, currently taking after 18 months

^a^Each twin brother had already started levothyroxine, as the NST revealed increased levels of TSH.

GA, gestational age; NST, newborn screening test; TFT, thyroid function test; fT4, free thyroxine; TSH, thyroid-stimulating hormone; SGA, small for gestational age; IVF, in vitro fertilization; C/S, caesarian section; Vag, vaginal delivery; MCDA, monochorionic diamniotic; DCDA, dichorionic diamniotic; DCTA, dichorionic triamniotic; MC, monochorionicity; ICM, iodine contrast media; TOF, tetralogy of Fallot; PA pulmonary atresia; PS, pulmonary stenosis; VSD, ventricular septal defect; ASD, atrial septal defect; PDA patent ductus arteriosus; PHT, pulmonary hypertension; DORV, double-outlet right ventricle; AVF, arteriovenous fistula; CT, computed tomography; MRI, magnetic resonance imaging

### Characteristics of the 12 infants who started levothyroxine medication

Table 2 presents the clinical characteristics of the 12 infants treated with levothyroxine. Of these, 4 (cases 1–4) of the 5 infants with v-TSH levels above 20 mU/L and 3 (cases 7–9) of 68 infants with v-TSH levels of 6–20 mU/L began receiving levothyroxine at the first repeat test. Excluding 7 patients (see white gray box in Fig 1) who had been administered levothyroxine and 737 infants with normal TSH values <6 mU/L at the first repeat test, 66 infants underwent the second repeat test (see white solid box in Fig 1) and 5 of these (cases 5, 6, 10, 11, and 12 in Table 2, and white gray solid box in Fig 1) began receiving levothyroxine treatment. One patient (case 5), whose v-TSH level was 21.7 mU/L following a contrast enema for meconium obstruction management, underwent retesting 2 weeks later and eventually began receiving medication at 150 days post birth due to persistent v-TSH elevation. Among the 12 patients who began receiving levothyroxine medication (Table 2), 2 died as a result of congenital anomalies, 5 successfully discontinued medication, and 5 under the age of 3 are still receiving treatment.

### Comparison between the normal TSH and dTSH groups

In comparison to the normal TSH group (n = 737), the dTSH group (n = 73) exhibited significantly higher TSH levels (6.6 ± 2.7 vs. 4.5 ± 2.0 mU/L for NST, and 10.9 ± 12.6 vs. 2.7 ± 1.4 mU/L for v-TSH levels at the first repeat test; p < 0.001 for both, Table 1). No significant differences were detected with regard to sex, monochorionicity, IVF pregnancy, or maternal thyroid disease between the normal TSH and dTSH groups. However, the dTSH group exhibited lower Bwt (2180.3 ± 499.2 vs. 2314.8 ± 376.5, p = 0.028) and higher proportions of Bwt <2,000 g (32.9% vs. 19.8%, p = 0.009), NICU admissions (39.7% vs. 18.0%, p < 0.001), congenital heart disease (17.8 vs. 5.7%, p = 0.001), congenital anomalies (23.3% vs. 4.2%, p < 0.001), exposure to ICM (13.7% vs. 0.3%, p < 0.001), and history of surgery (6.9% vs. 0.9%, p = 0.002) compared to the normal TSH group. Moreover, the dTSH group included a lower percentage of multiple births (71.2% vs. 84.1%, p = 0.005) and caesarian delivery (53.2% vs. 66.8%, p = 0.022) than did the normal TSH group (Table 1).

### Risk factors for dTSH

Among a total of 810 infants, a univariate regression analysis revealed that Bwt <2,000 g (p = 0.010), NICU admission (p < 0.001), singleton birth (p = 0.043 vs. twin births and p = 0.001 vs. triplet births), vaginal delivery (p = 0.024), congenital heart disease (p < 0.001), congenital anomaly (p < 0.001), exposure to ICM (p < 0.001), and history of surgery (p = 0.001) were significant risk factors for dTSH. A multivariate-adjusted model indicated that Bwt <2,000 g (OR 2.7, p = 0.002), triplet birth (OR 0.3, p = 0.015 vs. singleton), congenital anomaly (OR 4.1, p = 0.001), and exposure to ICM (OR 32.2, p < 0.001) were significant predictors for the occurrence of dTSH (Table 3). S2 Fig shows the robustness of selecting important variables in the models of 100 resampled data and distinct patterns of −2 × log-likelihood estimates with inclusion of covariates (LBW, multiple births, other congenital anomalies, and exposure to ICM) in the model. Other variables were not important for selection. The robustness of the variance explained by models with eight predictors was also observed. The R^2^ of the original data (0.15) was not significantly different from that of the bootstrapping data (95% confidence interval: 0.06–0.24). Therefore, the models predicting dTSH with eight predictors were stable regardless of the resampling data.

**Table 3:** Risk factors for delayed TSH elevation in 810 infants born at gestational ages of 35 and 36 weeks.

	Univariate		Multivariate	
	Odds ratio (95% CI)	p-value	Odds ratio (95% CI)	p-value
Low birth weight (<2,000 g)	2.0 (1.2–3.3)	0.010	2.7 (1.5–5.0)	0.002
NICU admission	3.0 (1.8–5.0)	<0.001	1.6 (0.9–3.1)	0.129
Multiple births				
Singleton	Reference		Reference	
Twin birth	0.6 (0.3–1.0)	0.043	0.9 (0.4–1.6)	0.636
Triplet birth	0.2 (0.1–0.5)	0.001	0.3 (0.1–0.8)	0.015
Monochorionicity	1.0 (0.5–1.0)	0.924		
Caesarian delivery (vs. vaginal delivery)	0.6 (0.4–1.0)	0.024	0.6 (0.4–1.1)	0.087
IVF pregnancy (vs. natural conception)	0.8 (0.5–1.3)	0.774		
Maternal thyroid disease	1.4 (0.7–2.7)	0.287		
Congenital heart disease (excluding PFO or PDA)	3.6 (1.8–7.1)	<0.001	0.8 (0.3–2.2)	0.699
Other congenital anomalies	6.7 (3.5–12.8)	<0.001	4.1 (1.8–9.4)	0.001
Exposure to iodine contrast media	58.3 (12.5–272.1)	<0.001	32.2 (5.3–196.1)	<0.001
History of surgery	7.7 (2.4–24.8)	0.001	0.6 (0.1–4.5)	0.614

TSH, thyroid-stimulating hormone; CI, confidence interval; NICU, neonatal intensive care unit; IVF, in vitro fertilization; PFO, patent foramen ovale; PDA patent ductus arteriosus

We additionally performed a subgroup analysis of 672 infants born as multiple (twin or triplet) births. Similarly, Bwt <2,000 g (OR 3.0, p = 0.002), congenital anomalies (OR 3.4, p = 0.022), and exposure to ICM (OR 37.6, p = 0.002) were positive predictors for the occurrence of dTSH, and a triplet birth (OR 0.3, p = 0.005 vs. twin births) was a negative predictor of dTSH (S2 Table). The models predicting dTSH with eight predictors in an analysis of 672 infants were also stable regardless of the resampling data (data not shown).

### Natural course of thyroid function in infants with v-TSH levels of 6–20 mU/L at the first repeat test

**Fig 2:**
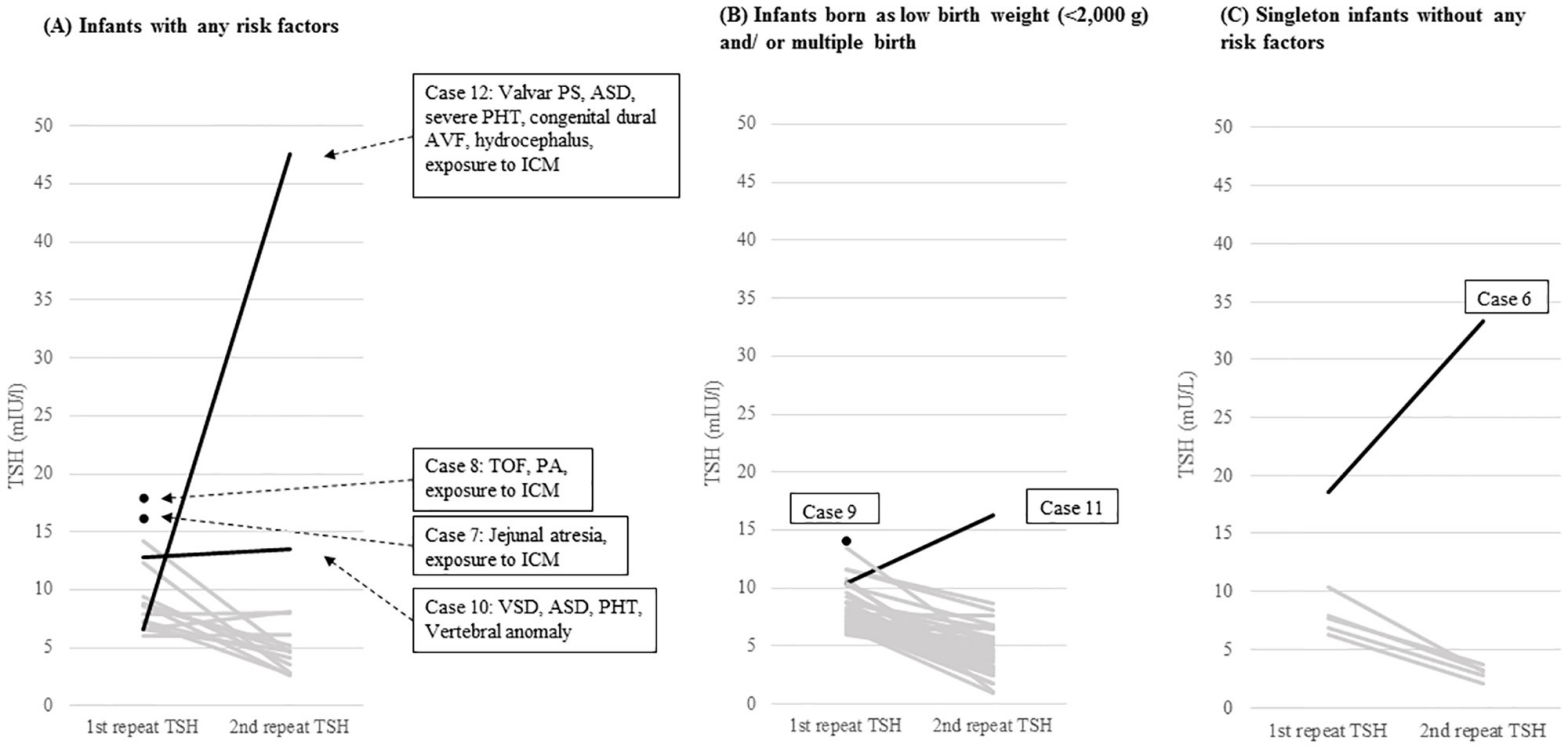
Changes in thyroid stimulating hormone (TSH) levels among 68 neonates with venous TSH levels of 6–20 mU/L at the first repeat test. (A) Infants with any risk factors (n = 20); (B) infants born at low birth weight and/or as a multiple birth without other risk factors (n = 42); and (C) singleton infants without any risk factors (n = 6) (four black lines indicate those starting levothyroxine after the second repeat test, three black spots indicate those starting levothyroxine after the first repeat test, and 61 gray lines indicate no treatment).

We categorized 68 infants whose v-TSH levels were 6–20 mU/L at the first repeat test (Fig 1) into three groups: 20 infants with risk factors including exposure to ICM and/or congenital heart defects or anomalies (Fig 2A), 42 LBW (Bwt <2,000 g) and/or multiple-birth infants without other risk factors (Fig 2B), and 6 singleton infants without any risk factors (Fig 2C). Fig 2A shows that 4 infants (case 7, 8, 10, and 12) started to take levothyroxine. In particular, case 12, who showed an abrupt increase in v-TSH level, from 6.6 to 47.5 mU/L, had a history of congenital heart disease, congenital dural fistula combined with hydrocephalus, and ICM exposure (Table 2). As shown in Fig 2B, case 9 started levothyroxine at a v-TSH level of 14.1 mU/L at the first repeat test, and case 11 began receiving levothyroxine treatment due to an increase in v-TSH level from 10.5 to 16.2 mU/L. The twins of both cases 9 and 11 had been already been treated with levothyroxine due to abnormal NST results, and they were excluded from this study according to the exclusion criteria shown in S1 Fig. In Fig 2C, case 6 started levothyroxine due to an increase in v-TSH level, from 18.5 to 33.4 mU/L (Table 2). Taken together, these results show that 6 of 16 infants with v-TSH levels of 10–20 mU/L were indicated for levothyroxine medication regardless of whether they had risk factors. However, among 52 infants with v-TSH levels of 6–10 mU/L, only 1 who had risk factors (case 12 in Fig 2A) was indicated for levothyroxine medication, and healthy late preterm infants exhibited normal thyroid function despite their being LBW and/or multiple birth infants (Fig 2B and 2C).

## Discussion

Seventy-three (9.0%) of 810 preterm neonates born at GA 35–36 weeks with normal NST results developed dTSH, defined as a v-TSH levels ≥6.0 mU/L at the first repeat test. Twelve (1.5%) of these began receiving treatment with levothyroxine. Independent predictors for the occurrence of dTSH included Bwt <2,000 g, a congenital anomaly, and exposure to ICM. When v-TSH levels were 6–20 mU/L at the first repeat test in infants born at GA 35–36 weeks, levothyroxine was more frequently indicated if infants exhibited risk factors. In those with a v-TSH level of 6–10 mU/L at the first repeat test, retesting should be done if they have risk factors; on the other hand, a second repeat test may not be necessary in healthy preterm infants.

Despite their normal NST results, 5 of the 810 infants had v-TSH levels ≥20 mU/L at the first repeat test, which indicated levothyroxine treatment, in accordance with the ESPE guidelines [2]. Furthermore, 2 patients exhibited low v-fT4 levels with significantly elevated TSH levels requiring prompt levothyroxine supplementation. Considering the importance of normal thyroid function to neurocognitive development, especially during the early years of life, a second screening test (the first repeat blood test in this study) is recommended for all preterm neonates. Causes of thyroid dysfunction among preterm infants include immaturity of the HPT axis continuing through 35 to 40 weeks gestation, immature thyroid hormone synthesis and metabolism, systemic diseases, and/or insufficient or excessive iodine intake [18, 19]. Therefore, in healthy late preterm infants, repeat specimens should be obtained to retest thyroid function.

The incidence of dTSH varied depending on subjects’ characteristics (i.e., GA, Bwt, presence of critical illness), the cutoff level for TSH elevation, and methodological differences. As the proportion of preterm infants born at low GA, VLBW infants, and those with risk factors such as critical illness, increases and the cutoff TSH level decreases, the incidence of dTSH may increase. Few studies have examined the frequency of dTSH occurrence among late preterm infants. Among preterm neonates born at 35 and 36 weeks and admitted to the NICU, dTSH frequency, defined as a TSH level >15 mU/L, was 12.3% and 6.3%, respectively [20]. When we included late preterm neonates born at GA 35–36 weeks and admitted to both the NB (80%) and the NICU (20%), the frequency of dTSH was 9.0%, although this included mild cases of dTSH, defined as a serum TSH level ≥6 mU/L at around 2 weeks (10–21 days) post birth. This study underlines the importance of a second screening test in late preterm infants, especially for those with a Bwt <2,000 g, a previous history of ICM exposure, or a congenital anomaly. A more attenuated HPT response and increased incidence of dTSH have been reported in extremely LBW or VLBW infants [2, 21]. Excess iodine temporarily inhibits thyroid hormone organification and synthesis via a mechanism known as “the acute Wolff-Chaikoff effect” [22]. Whereas the thyroid evades the acute Wolff-Chaikoff effect via downregulation of the thyrocyte sodium/iodide symporter in healthy individuals, when this evasion does not occur in preterm infants, iodine-induced hypothyroidism may ensue [23]. As ICM is frequently used for diagnostic imaging and therapeutic intervention, excess iodine exposure from ICM can disrupt thyroid function in preterm neonates [8, 24]. Although an increased risk for dTSH in infants delivered via caesarian section has been suggested due to placental transfer of topical iodide [25], caesarian section was not an independent predictor of dTSH in this study. Congenital malformations are detected more frequently among patients with CH than among control groups [10, 11]. Nine of the 12 infants requiring levothyroxine treatment in our study had congenital malformations (Table 2).

Our study population included high percentages of multiple births (83%), IVF pregnancies (65.7%), and caesarian deliveries (65.6%), as women at risk for preterm labor at GA 35–36 weeks caused by a high-risk pregnancy were transferred to our hospital because it is the tertiary referral center. A high prevalence of CH in multiple pregnancies has been reported [2, 26]. As the incidence of CH is more than three times higher among twins than among singleton births [26], a second screening at 2 weeks post birth is recommended, at least for same-sex twins, as fetal blood mixing between twins may have occurred [27]. Contrary to our expectations, a triplet birth was a negative predictor for dTSH compared to a singleton or twin birth, and IVF pregnancy was not independently associated with the occurrence of dTSH. Although a triplet birth was independently associated with dTSH in this study, risk factors (congenital heart disease, anomaly, history of ICM exposure, and surgery) associated with dTSH were less frequently observed in triplet births compared to singletons or twin births (S1 Table). Furthermore, as the proportion of singleton babies was too small to generalize this finding, further studies with large sample size are necessary to determine whether and why triplet birth is a negative predictor of dTSH. As hyperthyrotropinemia or subclinical primary hypothyroidism has been observed more frequently in IVF pregnancies than in those resulting from natural conception [28], the role of IVF in neonatal thyroid dysfunction remains to be further evaluated.

There is still no consensus as to whether neonates with v-TSH levels of 6–20 mU/L at a first repeat test should receive levothyroxine treatment or undergo a second repeat testing 2 weeks later [2]. In this gray area, clinicians should determine the management strategy considering the patient’s clinical information and risk factors. It is important to know the natural course of infants in this group according to other risk factors and not to overlook infants requiring levothyroxine. If a late preterm infant has risk factors related to thyroid dysfunction, such as a congenital anomaly and/or exposure to ICM, initiation of levothyroxine treatment or a second repeat test should be indicated in cases with v-TSH levels of 6–20 mU/L. However, if a late preterm infant is healthy, the second repeat test is indicated only for cases with v-TSH levels of 10–20 mU/L, whereas completion of retesting can be considered for cases with v-TSH levels of 6–10 mU/L. Nonetheless, cutoff levels should be determined based on long-term outcome data regarding clinical and laboratory results and physical and cognitive development derived from large numbers of infants. Since the primary role of screening is to prevent avoidable impairment by detecting hypothyroidism as early as possible, and even transient hypothyroidism during the critical stage of brain development in newborns can adversely affect long-term intellectual development [29], the threshold to complete retesting for healthy preterm neonates born at GA 35–36 weeks requires further evidence. Diagnostic imaging to establish definitive diagnoses is useful in distinguishing permanent CH from transient hyperthyrotropinemia. Even in patients who have commenced treatment, permanent hypothyroidism cannot be securely diagnosed without discontinuing treatment and retesting for normality by thyroid imaging. Moreover, the long-term outcomes of short-duration neonatal hyperthyrotropinemia, regardless of whether dTSH is transient or permanent, have not yet been elucidated [30, 31]. Since one longitudinal study reported that some newborns with “false positive” results at neonatal screening grew up with higher-than-normal levels of TSH during early childhood and had a risk of persistent subclinical hypothyroidism [32], monitoring of growth, development, and thyroid function is recommended for late preterm neonates with dTSH, particularly those with risk factors.

Our retrospective study had several limitations. As the study was conducted at a single tertiary referral center, selection bias was present due to the inclusion of mothers and infants at high risk for factors, such as multiple birth, IVF pregnancy, and congenital anomalies. Reproducibility may be limited depending on the proportion of high-risk infants. The long-term outcomes of the late preterm neonates with dTSH who received levothyroxine treatment could not be evaluated due to the short duration of the follow-up period. The lack of data on infants’ thyroid-disrupting medication or thyroid ultrasound and on maternal anti-thyroid antibodies was an additional limitation of this study. However, the study was strengthened by the inclusion and analysis of numerous late preterm infants born at GA 35–36 weeks, sufficient to investigate the frequency of and predictors for dTSH.

In conclusion, among preterm infants born at GA 35–36 weeks. dTSH was detected in 9.0% and levothyroxine supplementation was indicated for 1.5%. Even in the case of normal NST results, all late preterm infants born at GA 35–36 weeks should undergo a secondary screening test at around 2 weeks post birth. Bwt <2,000 g, exposure to ICM, and a congenital anomaly were identified as independent risk factors for dTSH. When v-TSH levels are ≥10 mU/L at the first repeat test, all late preterm infants need levothyroxine treatment or a second repeat test. When v-TSH levels are 6–10 mU/L, the second repeat test is indicated for late preterm infants with risk factors. Further studies are required using large numbers of infants to determine and support the threshold for healthy late preterm neonates.

## Supporting information

S1 Fig Flow diagram of the inclusion criteria for this study. (August 1, 2014–January 31, 2018).
(TIF)

S2 Fig Significance of model selection for covariates. (a) low birth weight, (b) NICU admission, (c) multiple birth, (d) caesarian delivery, (e) congenital heart disease, (f) other congenital anomalies, (g) exposure to iodine contrast media, and (h) history of surgery. Blue and red dots represent −2 × Log-likelihood according to the number of parameters in the generalized linear models without or with each covariate, respectively.
(TIF)

S1 Table Comparison of clinical and biochemical characteristics among singleton, twin, and triplet births. (DOCX)

S2 Table Risk factors for delayed TSH elevation in 672 infants born as a twin or triple at gestational ages of 35–36 weeks. (DOCX)
