# Supplementary figures and images for: How can the occurrence of delayed elevation of thyroid stimulating hormone in preterm infants born between 35 and 36 weeks gestation be predicted?
Source: PLoS One. 2019 Aug 23;14(8):e0220240. doi: 10.1371/journal.pone.0220240 (PMC6707626; doi:10.1371/journal.pone.0220240)

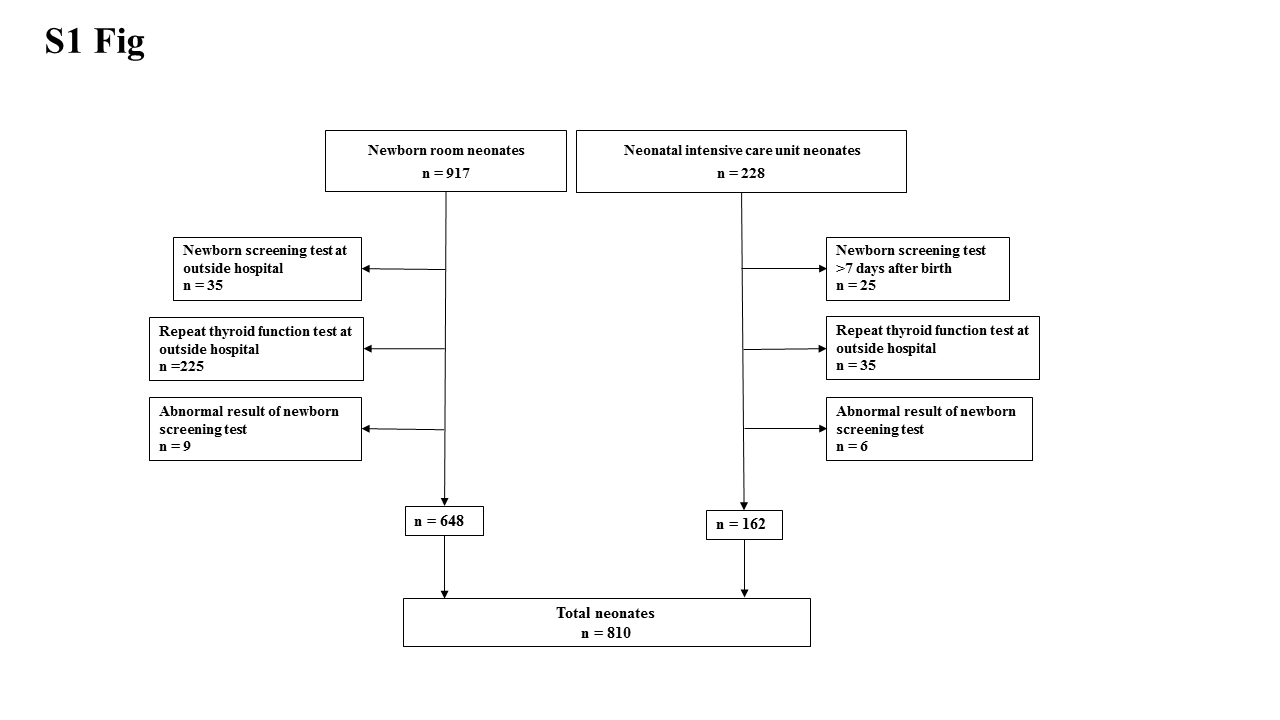

Supplement: S1 Fig — (August 1, 2014–January 31, 2018). (TIF) [file pone.0220240.s001.tif]

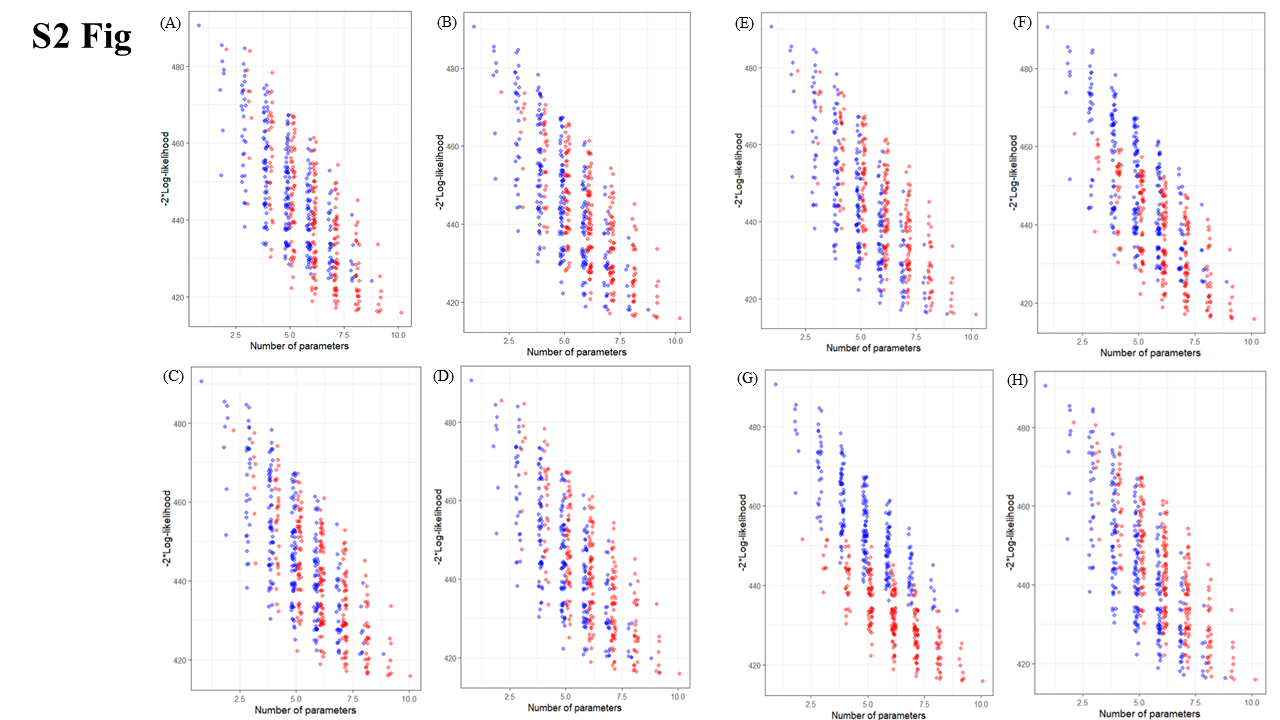

Supplement: S2 Fig — (a) low birth weight, (b) NICU admission, (c) multiple birth, (d) caesarian delivery, (e) congenital heart disease, (f) other congenital anomalies, (g) exposure to iodine contrast media, and (h) history of surgery. Blue and red dots represent −2 × Log-likelihood according to the number of parameters in the generalized linear models without or with each covariate, respectively. (TIF) [file pone.0220240.s002.tif]
