# Supplementary material for: How can the occurrence of delayed elevation of thyroid stimulating hormone in preterm infants born between 35 and 36 weeks gestation be predicted?
Source: PLoS One. 2019 Aug 23;14(8):e0220240. doi: 10.1371/journal.pone.0220240 (PMC6707626; doi:10.1371/journal.pone.0220240)
Supplement: S1 Table — (DOCX) [file pone.0220240.s003.docx]

**S1 Table. Comparison of clinical and biochemical characteristics among singleton, twin, and triplet births**

|  | Singleton  (n = 138) | Multiple birth(n = 672) | |
| --- | --- | --- | --- |
|  |  | Twin birth  (n = 491) | Triplet birth  (n = 181) |
| Boys, n (%) | 74 (53.6) | 241 (49.1) | 99 (54.7) |
| Low birth weight (<2,000 g), n (%)^a^ | 18 (13.0) | 74 (15.1) | 78 (43.1) |
| NICU admission, n (%) | 41 (29.7) | 84 (17.1) | 37 (20.4) |
| Monochorionicity, n (%) |  | 104 (21.2) | 26 (14.4) |
| Caesarian delivery, n (%)^a^ | 84 (60.9) | 293 (59.7) | 27 (85.1) |
| IVF pregnancy, n (%)^a^ | 27 (19.6) | 148 (69.9) | 19 (89.5) |
| Maternal age at pregnancy (years) | 33.8 ± 4.5 | 33.0 ± 3.3 | 34.0 ± 3.4 |
| Maternal thyroid disease, n (%) | 15 (10.9) | 79 (16.1) | 17 (2.1) |
| Congenital heart disease excluding PFO or PDA, n (%)^a^ | 18 (13.0) | 32 (6.5) | 5 (2.8) |
| Other congenital anomalies, n (%)^a^ | 22 (15.9) | 23 (4.7) | 4 (2.2) |
| Exposure to iodine contrast media, n (%)^b^ | 7 (5.1) | 4 (0.8) | 1 (0.6) |
| History of surgery, n (%)^b^ | 7 (5.1) | 4 (0.8) | 1 (0.6) |

Data are expressed as mean ± standard deviation (SD) or number (%).

TSH, thyroid stimulating hormone; NICU, neonatal intensive care unit; IVF, *in vitro* fertilization; PFO, patent foramen ovale; PDA, patent ductus arteriosus; NST, newborn screening test; fT4, free T4

^a^*P* < .001 using the χ2 test or ANOVA for trends across groups.

^b^*P* < .01 using the χ2 test for trends across groups
