# Supplementary material for: How can the occurrence of delayed elevation of thyroid stimulating hormone in preterm infants born between 35 and 36 weeks gestation be predicted?
Source: PLoS One. 2019 Aug 23;14(8):e0220240. doi: 10.1371/journal.pone.0220240 (PMC6707626; doi:10.1371/journal.pone.0220240)
Supplement: S2 Table — (DOCX) [file pone.0220240.s004.docx]

**S2 Table.** **Risk factors for delayed TSH elevation in 672 infants born as twins or triplets at gestational ages between 35 and 36 weeks**

|  | Univariate | | Multivariate | |
| --- | --- | --- | --- | --- |
|  | Odds ratio (95% CI) | p-value | Odds ratio (95% CI) | p-value |
| Low birth weight (<2,000 g) | 2.5 (1.4–4.5) | 0.002 | 3.0 (1.5–5.8) | 0.002 |
| NICU admission | 0.3 (0.2–0.6) | <0.001 | 1.9 (0.9–3.9) | 0.067 |
| Triplet birth (vs. twin) | 0.4 (0.2–0.9) | 0.027 | 0.3 (0.1–0.7) | 0.005 |
| Monochorionicity | 1.3 (0.6–2.5) | 0.479 |  |  |
| Caesarian delivery (vs. vaginal delivery) | 0.7 (0.4–1.2) | 0.163 |  |  |
| IVF pregnancy (vs. natural conception) | 0.8 (0.4–1.5) | 0.488 |  |  |
| Maternal thyroid disease | 1.5 (0.7–3.1) | 0.291 |  |  |
| Congenital heart disease (excluding PFO or PDA) | 2.5 (0.4–6.2) | 0.054 | 0.7 (0.2–2.5) | 0.629 |
| Other congenital anomalies | 4.7 (1.9–11.6) | 0.001 | 3.4 (1.2–10.0) | 0.022 |
| Exposure to iodine contrast media | 51.6 (5.7–470.6) | <0.001 | 37.6 (3.8–368.8) | 0.002 |
| History of surgery | 3.0 (0.3–27.5) | 0.327 |  |  |

*P*-value was calculated for comparison between the normal group and delayed TSH elevation group

TSH, thyroid-stimulating hormone; CI, confidence interval; NICU, neonatal intensive care unit; IVF, in vitro fertilization; PFO, patent foramen ovale; PDA patent ductus arteriosus
